# Validation of the Italian version of the intuitive exercise scale: a four-factor structure in the general population

**DOI:** 10.1007/s40519-025-01718-3

**Published:** 2025-02-03

**Authors:** Paolo Mancin, Marta Ghisi, Tatiana Moro, Silvia Cerea

**Affiliations:** 1 Department of General Psychology, University of Padova, Via Venezia, 8, 35131 Padua, Italy; 2 U.O.C. Hospital Psychology, University-Hospital of Padua, Padua, Italy; 3 Department of Biomedical Sciences, University of Padua, Padua, Italy

**Keywords:** Intuitive exercise, Italian validation, Physical activity, Intuitive eating, Exercise addiction

## Abstract

**Purpose:**

Intuitive exercise may foster healthier engagement in physical activity, leading to enhanced well-being and more intuitive eating habits. The Intuitive Exercise Scale (IEXS) was originally developed to assess this construct in English speakers. The IEXS is composed of four scales addressing distinct facets of intuitive exercise: emotional exercise, body trust, exercise rigidity, mindful exercise. The present study aimed to validate an Italian translation of the IEXS, exploring its factorial structure, invariance, internal consistency, and convergent validity.

**Methods:**

A sample of 1140 women and men (age: *M* = 45.25, SD = 11.64, range 22–76 years) was recruited from the Italian general population; 61.8% were physically active. Inclusion criteria were being an Italian native speaker and being at least 18 years. The sample was randomly split in two equal halves for the Exploratory Factor Analysis (*n* = 570; women: 50%; physically active: 61.8%) and the Confirmatory Factor Analysis (*n* = 570; women: 50%; physically active: 61.8%). Multi-group confirmatory factor analyses were performed to assess invariance among women and men and physically and non-physically active individuals. McDonald’s omega was used to assess internal consistency. Pearson’s correlations were utilized to assess convergent validity.

**Results:**

The Italian IEXS replicated a four-factor structure and showed good invariance among women and men. Invariance among physically and non-physically active individuals was only partially supported. Excellent internal consistency and adequate convergent validity were achieved.

**Conclusions:**

The IEXS demonstrated to be a valid measure to examine intuitive exercise in the Italian population, replicating and expanding the findings of the English version.

*Level of evidence* Level V, Cross-sectional, Psychometric study.

**Supplementary Information:**

The online version contains supplementary material available at 10.1007/s40519-025-01718-3.

## Introduction

In recent years, scholars aimed to identify possible positive attitudes toward physical activity that could be incorporated in treatments of individuals with eating disorders [1–3]. Indeed, dysfunctional exercise patterns are found in people with dysfunctional eating behaviors [4]. For this purpose, some scholars have introduced the concept of intuitive exercising [2, 5]. This construct is divided into four distinct dimensions: body trust, which involves following body cues to guide the type, frequency, and intensity of physical activity; mindful exercise, which entails being aware of how the body feels while moving; exercise rigidity, which involves engaging in a range of physical activities; and emotional exercise, which relates to choosing physical activities to regulate negative emotions and promote positive ones [5, 6]. Several studies, conducted with samples from both general and clinical populations, have examined this construct, highlighting its promising role in fostering individuals’ well-being and functional eating behaviors [5–8].

The Intuitive Exercise Scale (IEXS) was developed to examine this construct. In an English-speaking community sample of men and women, the IEXS demonstrated a four-factor structure, gender invariance, and appropriate convergent validity [5]. In a later study, a three-factor structure was a better fit in a sample of women with eating disorders, with the body trust and mindful exercise dimensions merging into a single factor named “body intuition” [6]. These differing findings might raise questions about the generalizability of the results and how intuitive exercise is experienced across populations. Accordingly, previous research on the validation of psychological instruments in Italy, such as Diotaiuti et al.’s [9] work on the Communication Styles Inventory, highlights the need for culturally adapted scales to ensure measurement accuracy and reliability in diverse populations. Hence, the use of the IEXS should be further assessed in various contexts and populations.

In the current study, we aimed to explore the factorial structure of the IEXS in a sample of Italian individuals from the general population. Our objectives were to identify the most appropriate factorial structure and evaluate invariance among men and women, as well as between physically and non-physically active individuals. Finally, we aimed to examine convergent validity with measures assessing exercise addiction symptoms and intuitive eating dimensions.

## Methods

### Participants

The main sample comprised 1140 participants (50% women): their age ranged between 22 and 76 years (M = 45.25, SD = 11.64). 704 participants reported to be physically active. Inclusion criteria were being an Italian native speaker and being at least 18 years.

The main sample was randomly divided in two halves (n = 570): the first half was used for the Exploratory Factor Analysis (EFA), while the second half was used for the Confirmatory Factor Analysis (CFA) and invariance measurement.

### Measures

Participants self-reported sociodemographic information, including age, sex, gender, years of education, marital status, occupation, and current psychological disorders. Involvement in physical activity was assessed using a self-report item with a dichotomous response format (yes/no), asking “Do you practice physical activity/physical exercise/sport?”.

To prepare the Italian version of the IEXS, the translation process followed the standard steps proposed in the psychology literature [10]. Three independent translators, with broad expertise in sport sciences and clinical psychology, translated each item into Italian. The main challenge encountered was avoiding technical terms and selecting appropriate wordings that would facilitate the item interpretation for readers with diverse educational levels and various levels of engagement with physical activity. Then, the translators reached an agreement on a shared Italian translation of the IEXS. The shared version was back-translated in English by a bilingual individual. The back-translated version was compared to the original one, highlighting only minor adjustments required to reach a final Italian version of the IEXS. The items of the Italian translation of the IEXS are presented in the Supplementary Material (S-Table 1).

The Intuitive Exercise Scale (IEXS) is a 14-item questionnaire measuring intuitive exercise. The questionnaire is composed of four subscales: Emotional Exercise, Body Trust, Exercise Rigidity, and Mindful Exercise. The items are rated on a 5-point scale ranging from 1 (“strongly disagree”) to 5 (“strongly agree”). Higher score indicates higher tendencies toward intuitive exercise. The original version of the questionnaire showed good internal consistency in all the four subscales (Composite Reliability indices ranging from 0.74 to 0.88) and good construct validity [5].

The Exercise Addiction Inventory (EAI) was employed to assess exercise addiction symptoms, which is defined through the components of the addiction model: tolerance, withdrawal, salience, conflict, mood modification, relapse. This self-report measure is composed of 6 items and a 5-point Likert scale ranging from 1 (“strongly disagree”) to 5 (“strongly agree”). Higher scores represent higher risk of exercise addiction [11–13]. Only physically active participants were asked to complete this measure. In the current study, McDonald’s ω was 0.75.

The Intuitive Eating Scale-2 (IES-2) measures intuitive eating attitudes and behaviors. The self-report questionnaire is composed of 23 items rated on a 5-point Likert scale, ranging from 1 (“strongly disagree”) to 5 (“strongly agree”). It enables to compute a total score as well as four scores: Unconditional Permission to Eat, Eating for Physical Rather than Emotional Reasons, Reliance on Hunger and Satiety Cues, and Body-Food Choice Congruence. Higher scores are representative of engagement in intuitive eating practices [14, 15]. In the present study, internal consistency was appropriate for the total score (McDonald’s ω = 0.92) and each subscale (McDonald’s ωs ranging from 0.76 to 0.90).

### Procedures

Participants were recruited through advertisements posted on social media platforms and with fliers distributed around the University of Padova. Participants were provided with a link to access an online survey, which included an informed consent followed by the self-report measures.

### Statistical analysis

The dataset was randomly split into equal halves for the EFA and the CFA. Randomization was conducted with the raw data. Equal distributions of women and men (women: 285; men: 285) and of physically and non-physically active individual (physically active: 352; non-physically active: 218) were pursued for each half of the sample. An equal distribution would have allowed us to have two representative subsamples for the analyses. The randomization process was repeated until the chosen distribution was reached.

The Kaiser–Meyer–Olkin (KMO) should be at least ≥ 0.70 and Bartlett’s test of sphericity should be significant to justify the application of an EFA [16]. Eigenvalues (> 1) and a parallel analysis identified the appropriate number of factors. Both the EFA and the CFA were conducted utilizing a Maximum Likelihood estimation; an oblimin rotation was adopted for the EFA. The model chi-square (χ^2^), the Comparative Fit Index (CFI), the Standardized Root Mean Square Residual (SRMR), the Root Mean Square Error of Approximation (RMSEA) and its 90% Confidence Interval (CI), the Tucker–Lewis Index (TLI), and the Bayesian Information Criterion (BIC) were used to assess the models. The CFI should show values ≥ 0.95 for adequate fit, the SRMR should show values < 0.09 for good fit, the RMSEA could show values up to 0.08 for an adequate fit, and the TLI should show values ≥ 0.95 for good fit [17]. Items’ factor loadings were considered appropriate when > 0.30.

To test for invariance, multi-group CFAs were performed considering men and women and physically and non-physically active individuals. A chi-square (χ^2^) was utilized to test invariance by comparing the constrained with the unconstrained model.

Internal consistency was assessed utilizing McDonald’s ω coefficient [18]: values should be greater than 0.70 for adequacy [19].

Finally, we tested convergent validity employing Pearson’s correlations among the IEXS dimension, the IES-2 scores, and the EAI score. According to Cohen [20], correlations ≤ 0.10 were considered week, ~ 0.30 were considered moderate, and ~ 0.50 were considered strong.

## Results

Utilizing the first half of the sample, the KMO (0.85) and Bartlett’s test of sphericity (*p* < 0.001) were adequate to perform an EFA. Both the examination of eigenvalues and the parallel analysis demonstrated 4 factors as the proper number. We decided to compare this solution with a three-factor one. The four-factor structure (*χ*^2^(41) = 129.92; *p* < 0.001; RMSEA = 0.06 (90% CI 0.05, 0.07); TLI = 0.97; BIC = − 130.25) was preferred over the three-factor structure (*χ*^2^(52) = 970.23; *p* < 0.001; RMSEA = 0.18 (90% CI 0.17, 0.19); TLI = 0.71; BIC = 640.26). Factor loadings are presented in Table 1.

**Table 1:** Findings from the Exploratory Factor Analysis with three and four factors and the Confirmatory Factor Analysis with four factors

Items	EFA with three factors			EFA with four factors				CFA with four factors
	Factor 1	Factor 2	Factor 3	Factor 1 Emotional exercise	Factor 2 Exercise variety	Factor 3 Body trust	Factor 4 Mindful Exercise	
1	− 0.01	0.04	**0.72**	0.01	0.06	− 0.02	**0.72**	**0.69**
2	**0.84**	− 0.02	0.01	**0.84**	− 0.02	< 0.01	0.01	**0.82**
3	**0.74**	0.02	− 0.08	**0.75**	0.05	− 0.05	− 0.05	**0.80**
4	0.12	**0.40**	0.24	0.01	0.01	**0.85**	< 0.01	**0.88**
5	0.12	**0.40**	0.27	< 0.01	− 0.04	**0.93**	< 0.01	**0.87**
6	< 0.01	− 0.04	**0.93**	0.02	− 0.02	− 0.01	**0.96**	**0.93**
7	0.12	**0.45**	0.25	0.01	0.07	**0.82**	0.02	**0.87**
8	**0.92**	− 0.02	0.03	**0.92**	− 0.01	< 0.01	0.03	**0.86**
9	**0.73**	< 0.01	− 0.06	**0.73**	− 0.02	0.05	− 0.07	**0.74**
10	0.02	**0.87**	− 0.06	0.03	**0.79**	0.12	− 0.07	**0.91**
11	− 0.03	− 0.01	**0.87**	− 0.03	− 0.01	0.04	**0.85**	**0.89**
12	− 0.05	**0.93**	− 0.01	− 0.04	**0.94**	0.01	0.02	**0.89**
13	0.02	**0.84**	− 0.02	0.05	**0.89**	− 0.07	0.03	**0.88**
14	**0.85**	0.03	0.02	**0.85**	0.02	0.01	0.02	**0.82**

The EFA was conducted with Maximum Likelihood and an oblique rotation. The CFA was conducted with Maximum Likelihood estimation. Factor above .30 are in bold

We tested the four-factor structure emerged in the EFA through a CFA with the second half of the sample (Table 1). The model demonstrated good fit (*χ*^2^(71) = 249.49, *p* < 0.001; CFI = 0.97; TLI = 0.96; RMSEA = 0.07 [90% CI 0.06, 0.08]; SRMR = 0.04). All items loaded positively and significantly (*p* < 0.001) in the intended factors. The four factors extracted confirmed the model of Intuitive Exercise and its four components: emotional exercise, body trust, exercise variety, and mindful exercise. Opposite to the original English version, the items constituting the exercise rigidity subscale demonstrated positive factor loadings. Hence, the subscale was named “exercise variety”.

The four factors also demonstrated excellent internal consistency: emotional exercise (*ω* = 0.90), body trust (*ω* = 0.91), exercise variety (*ω* = 0.92), and mindful exercise (*ω* = 0.87).

The four-factor structure was adequate for both women and men at configural level (*χ*^2^(142) = 346.79, *p* < 0.001; CFI = 0.96; TLI = 0.95; RMSEA = 0.07; SRMR = 0.04). Invariance was also proved constraining factor loadings (Δ*χ*^2^(10) = 7.06, *p* = 0.72) and intercepts (Δ*χ*^2^(10) = 16.91, *p* = 0.08). Moreover, the four-factor structure was appropriate for both physically and non-physically active individuals at configural level (*χ*^2^(142) = 360.40, *p* < 0.001; CFI = 0.96; TLI = 0.95; RMSEA = 0.07; SRMR = 0.04). However, the model did not show invariance after constraining factor loadings (Δ*χ*^2^(10) = 46.88, *p* < 0.001). The factor loadings of four items (items 11, 6, 13, and 7) should be estimated freely to reach partial metric invariance (Δ*χ*^2^(6) = 8.17, *p* = 0.23), highlighting possible differences in how the construct of intuitive exercise is experienced.

As for convergent validity (Table 2), correlations were computed separately for physically and non-physically active individuals. As expected, the subscales body trust and mindful exercise showed positive correlations with the dimensions of intuitive eating. The subscales body trust, exercise variety, and mindful exercise showed moderate-to-weak correlations with the subscales Eating for Physical Rather than Emotional Reasons, Reliance on Hunger and Satiety Cues, and Body-Food Choice Congruence of the IES-2. The emotional exercise subscale demonstrated moderate-to-weak correlations with the subscales Unconditional Permission to Eat and Body-Food Choice Congruence of the IES-2. Among physically active individuals, the EAI total score showed moderate-to-weak correlations with each scale of the IEXS, except for the emotional exercise scale, which was strongly correlated.

**Table 2:** Means, Standard Deviations, and Correlations between Intuitive Exercise, Intuitive Eating, and Exercise Addiction divided for physically and non-physically active individuals

	1	2	3	4	5	6	7	8	9	10	Mean (SD)
1. IEXS: emotional exercise		0.30***	0.49***	0.10*	− 0.12*	− 0.30***	− 0.02	− 0.06	0.11*	/	2.07 (.89)
2. IEXS: body trust	0.19***		0.47***	0.33***	0.27***	0.01	0.18***	0.32***	0.31***	/	2.74 (1.05)
3. IEXS: exercise variety	0.29***	0.34***		0.23***	0.17***	− 0.06	0.19***	0.15**	0.23***	/	2.29 (.96)
4. IEXS: mindful exercise	− 0.19***	0.29***	− 0.03		0.17***	< 0.01	0.12*	0.20***	0.21***	/	3.47 (1.02)
5. IES-2 total score	− 0.09*	0.33***	0.25***	0.21***		0.54***	0.83***	0.83***	0.45***	/	3.44 (.57)
6. IES-2: Unconditional Permission to Eat	− 0.20***	0.08*	0.01	0.15***	0.55***		0.18***	0.28***	− 0.05	/	3.60 (.78)
7. IES-2: Eating for Physical Rather than Emotional Reasons	− 0.11**	0.15***	0.20***	0.10*	0.82***	0.20***		0.57***	0.27***	/	3.33 (.84)
8. IES-2: Reliance on Hunger and Satiety Cues	− 0.01	0.48***	0.22***	0.26***	0.81***	0.30***	0.48***		0.41***	/	3.50 (.80)
9. IES-2: Body-Food Choice Congruence	0.22***	0.30***	0.34***	0.07	0.46***	− 0.09*	0.26***	0.48***		/	3.28 (.78)
10. EAI total score^1^	0.51***	0.10**	0.24***	− 0.27***	− 0.11**	− 0.30***	− 0.05	− 0.03	0.18***		/
Mean (SD)	2.81 (.95)	3.26 (.90)	3.37 (.96)	3.50 (.90)	3.54 (.55)	3.41 (.73)	3.54 (.85)	3.61 (.77)	3.64 (.74)	15.34 (4.81)	

1 = the Exercise Addiction Inventory was completed only by physically active individuals

SD = Standard Deviation; IEXS = Intuitive Exercise Scale; IES-2 = Intuitive Eating Scale-2; EAI = Exercise Addiction Inventory. Means, standard deviations, and correlations of physically active individuals are below diagonal; means, standard deviations, and correlations of non-physically active individuals are above diagonal

^*^p < .05

**p < .01

***p < .001

## Discussion

The Italian translation of the IEXS supported the presence of four factors with adequate internal consistency, consistent with findings from an English-speaking community sample and with some initial findings of this version [5, 21]. This study further confirmed a four-factor structure for the IEXS in community samples. However, contrary to the original English version, the items composing the exercise rigidity subscale demonstrated positive factor loadings, as found with a clinical sample [6]. Thus, the subscale was named “exercise variety” in this version: higher scores are indicative of flexibility/intuitiveness with physical exercise.

Gender invariance was replicated. Thus, the Italian version of the IEXS enables to achieve reliable score comparisons between women and men. Interestingly, despite demonstrating an appropriate factor structure, the Italian IEXS showed only partial invariance among physically and non-physically active individuals. This finding suggested that individuals may experience intuitive exercise regardless of their involvement in physical activity. They may be attuned to their internal signals, guiding their engagement in physical activity, as it happens with intuitive eating [22]. However, the extent of involvement in physical activity might shape the experience of intuitive exercise differently, complicating group comparisons. According to the current findings, score comparisons between physically and non-physically active individuals should be interpreted with great caution. Future studies should further explore this issue.

Findings pertaining the correlations between intuitive exercise dimensions and exercise addiction symptoms provided new insights. The weak negative correlation between mindful exercise and exercise addiction supported the role of intuitive exercise as an adaptive approach to physical activity. In contrast to the previous validation study, exercise addiction symptoms demonstrated a strong positive association with emotional exercise and a weak positive association with exercise variety [5]. These findings highlighted potential similarities between intuitive exercise and exercise addiction: Using physical activity to regulate negative emotions could be a shared feature of both constructs. Furthermore, engaging in various forms of physical activity could indicate either flexibility or excessive engagement in physical activity. Future studies should further investigate the overlap and shared features between these constructs.

Consistent with the study by Reel et al. [5], the dimensions of body trust and mindful exercise demonstrated correlations with dimensions of intuitive eating. Interestingly, the emotional exercise dimension demonstrated both a positive correlation with body-food choice congruence and a moderate negative correlation with the dimension of unconditional permission to eat. The dual nature of the emotional exercise dimension, indicated by its associations with exercise addiction symptoms, may help explain these associations. As for the exercise variety dimension, positive associations with intuitive eating were found, which aligned with the conceptualization of intuitive exercise [5].

### Strength and limits

This study had some limitations. We did not collect information about specific types of physical activity performed or the amount of time spent on physical activity. Moreover, we did not evaluate temporal stability of the IEXS. The sample of non-physically active individuals is lower in numerosity compared to the sample of physically active ones. Thus, generalizability and reliability of the findings to the former population may be undermined. Reaching only partial metric invariance between physically and non-physically active individuals could be a limitation in the applicability of the IEXS in the broader population. Hence, future studies should further evaluate invariance by, for instance, increasing the sample size of non-physically active individuals and ensuring a more balanced distribution. As another limitation, possible nuances of the original English version of the IEXS may be lost during the Italian translation of the IEXS, consequently influencing the findings. For instance, the cultural adaptation of the IEXS might be responsible of the different findings pertaining to the exercise rigidity/variety dimension.

Future studies should further explore potential nuances in the experience of intuitive exercise, especially among individuals with different levels of physical activity engagement. Moreover, a comprehensive examination of the emotional regulation dimension seems warranted.

The main strength of this study was the reliance on an overall large sample of Italian individuals. Moreover, the current study employed a rigorous explorative method to examine the factorial structure of the IEXS, adopting both an EFA and a CFA. The sample size enabled also to conduct multigroup CFA to further explore the invariance of the IEXS comparing women and men, and physically and non-physically active individuals.

## Conclusions

The Italian translation of the IEXS emerged as a valid measure to examine intuitive exercise in the Italian population. Moreover, the current findings provided useful insights on the use of this measure in the general population and suggested further examination of this measure in diverse samples (e.g., individuals engaging in different levels of physical activity).

### What is already known on this subject?

- The IEXS assesses intuitive exercise, a novel construct that enables to achieve a functional relation with physical exercise.
- The IEXS demonstrated a four-factor structure in an English-speaking community sample, while a three-factor structure was preferred for individuals with eating disorders. Moreover, the IEXS showed good internal consistency and convergent validity.
- The IEXS showed good invariance considering women and men.

### What this study adds?

- This study provided further support for a four-factor structure of the IEXS in a community sample of Italian speakers.
- The good psychometric properties of the IEXS were further supported.
- This study is the first to examine the invariance of the IEXS among physically and non-physically active individuals, highlighting possible differences in how this construct is experienced.
- The associations between the emotional exercise dimension of intuitive exercise and exercise addiction shed light on possible connections between these two constructs, which require further examination.

## Supplementary Information

Below is the link to the electronic supplementary material.

Supplementary file 1.

## Data Availability

The datasets generated during and/or analysed during the current study are available from the corresponding author on reasonable request.
