# Supplementary material for: Validation of the Italian version of the intuitive exercise scale: a four-factor structure in the general population
Source: Eat Weight Disord. 2025 Feb 3;30(1):12. doi: 10.1007/s40519-025-01718-3 (PMC11790799; doi:10.1007/s40519-025-01718-3)
Supplement: Supplementary file 1 [file 40519_2025_1718_MOESM1_ESM.docx]

|  | **Italian translation of the IEXS items** |
| --- | --- |
| 1. | Smetto di praticare esercizio fisico quando sento dolore. |
| 2. | Mi capita di praticare esercizio fisico quando provo emozioni negative (ad esempio, quando mi sento ansioso/a, depresso/a, triste) anche se non me la sento di praticarlo. |
| 3. | Mi capita di praticare esercizio fisico quando sono solo/a, anche se non me la sento di praticarlo. |
| 4. | Ho fiducia nel fatto che il mio corpo mi dica quando praticare esercizio fisico |
| 5. | Ho fiducia nel fatto che il mio corpo mi dica quale tipo di esercizio fisico praticare. |
| 6. | Smetto di praticare esercizio fisico quando mi sento affaticato/a. |
| 7. | Ho fiducia nel fatto che il mio corpo mi dica quanto esercizio fisico praticare. |
| 8. | Uso l’esercizio fisico per avere un aiuto nell’alleviare le mie emozioni negative. |
| 9. | Mi capita di praticare esercizio fisico quando sono stressato/a, anche se l’ho già praticato. |
| 10. | Includo differenti tipologie di attività fisica nel mio programma di esercizio fisico. |
| 11. | Quando sento che il mio corpo è stanco, smetto di praticare esercizio fisico. |
| 12. | Mi piace praticare differenti tipologie di attività fisica quando pratico esercizio fisico. |
| 13. | Pratico diverse tipologie di esercizio fisico |
| 14. | Uso l’esercizio fisico per distrarmi dalle emozioni negative o per evitarle. |

**S-Table 1.**

*The items of the IEXS in their Italian translation.*
